# Effects of exercise in cool versus hot conditions on pathways of gut permeability, systemic inflammation, and iron homeostasis in female athletes

**DOI:** 10.14814/phy2.70981

**Published:** 2026-06-17

**Authors:** Kathryn M. Lucernoni, Samantha J. Chacon, Karen Wiedenfeld Needham, John R. Halliwill, Christopher T. Minson

**Affiliations:** ^1^ Bowerman Sports Science Center, Department of Human Physiology University of Oregon Eugene Oregon USA

**Keywords:** endotoxins, heat exposure, hepcidin, inflammation, interluekin‐6, lipopolysaccharide

## Abstract

The purpose of this study was to examine the time course and magnitude of the inflammatory, hepcidin and endotoxin responses evoked by exercise under hot environmental conditions in female athletes. In a randomized cross‐over study design, 13 endurance trained female athletes (age 26 (range: 20–42) years; VO_2_max 57.7 (53.4, 62.1) mL·kg^−1^·min^−1^; mean (95% CI)) completed two exercise sessions on a treadmill at 70% VO_2_max in hot (35°C and 40% RH) and cool (11°C and 40% RH) environmental conditions. Peak body core temperature during the hot condition was greater than the cool condition (change +2.1°C (1.8, 2.3); +1.0°C (0.8, 1.3); *p* < 0.01). There were no changes in LPS or TNFα concentrations at any time point. The hot condition had a greater rise in IL‐6 concentration at 1 h post exercise (hot: 1.6 pg/mL (1.0, 2.2); cool: 1.0 pg/mL (0.8, 1.3); *p* = 0.04). Hepcidin concentrations at 3 h post exercise were greater in the hot condition (hot: 15.1 ng/mL (8.8, 21.3); cool: 7.9 ng/mL (3.5, 12.2); *p* = 0.03). These data suggest that moderate exercise in a hot environment evokes a greater IL‐6 concentration and hepcidin concentration post exercise than a cool environment without influence of gut permeability.

## 1 INTRODUCTION

Iron is an essential mineral, critical for many physiological processes and athletic performance. The athletic population is particularly susceptible to poor iron status with female athletes having a significantly higher prevalence of iron deficiency compared to males (35%, 10%; respectively) (Fallon, 2008; Sim et al., 2019). Further, 60% of elite collegiate female athletes experienced anemia during their career (Cowell et al., 2003). If iron consumption and absorption from the diet is not adequate and/or chronic stressors occur, iron stores can be depleted. Iron can be lost during exercise via hemolysis, hematuria, sweat, menstrual bleeding, and gastrointestinal bleeding (Babić et al., 2001; DeRuisseau et al., 2002; Mclnnis et al., 1998; Zoller & Vogel, 2004). The labile iron pool can be increased by dietary iron absorption. However, there are obstacles that can make iron absorption from the diet difficult. Iron absorption is stringently regulated as humans have no excretory pathway to remove excess iron, relying on blocking absorption of ingested iron from the intestines or sequestering iron within cells. Hepcidin is the key iron‐regulating hormone. Hepcidin functions by blocking the only known cellular iron‐exporting channel (ferroportin) leading to reduced iron availability in circulation and reduced absorption of dietary iron (Ganz & Nemeth, 2012). If hepcidin is high, then the ability to absorb iron to increase iron stores is limited (Barney et al., 2022).

Exercise is a large stimulus for release of hepcidin due to the post exercise inflammatory release of cytokines. Interluekin‐6 (IL‐6) has been identified as an important inflammatory and energy sensing cytokine that regulates production of hepcidin (Lee et al., 2005; Nemeth et al., 2004; Nemeth & Ganz, 2009). Previous research has established an increase in hepcidin hormone concentration after exercise across different modalities and intensities (Sim et al., 2013). Hepcidin concentration peaks ~3 h after an acute exercise session (Peeling et al., 2009) and can remain elevated for >12 h after exercise. Due to the nature of competitive athlete training, this indicates that hepcidin can be chronically elevated, making it difficult to absorb dietary iron (Ishibashi et al., 2022; Peeling, 2010). Hepcidin has been implicated as a main mechanism that leads to iron deficiency in athletes (Peeling et al., 2009). However, much of the preliminary work on exercise and hepcidin was conducted on male athletes with limited focus on female athletes (Ishibashi et al., 2017, 2022), despite females being more susceptible to iron deficiency. Further, there is limited consideration for how environmental stimuli may impact this response. Seasonal changes in ambient training temperature and competitions align within warmer months (Barney et al., 2022), meaning athletes may be more susceptible to issues with iron regulation during this time. Exercise in hot environmental conditions can result in higher IL‐6 production due to changes in relative exercise intensity (McKay et al., 2021) and previous work in a field‐based investigation showed a positive correlation between ambient temperature and hepcidin concentration (McKay et al., 2020). Only one controlled study of iron regulation post exercise in the heat has been done on male athletes resulting in greater elevations in IL‐6, but not hepcidin in the hot ambient conditions (McKay et al., 2021). However, females may have a different response due to baseline ferritin (Peeling et al., 2017). Hepcidin concentration is reliant on serum ferritin which tends to be lower in the female population and therefore may drive a different response. There is a gap in the literature examining female athletes and issues pertinent to them (Costello et al., 2014; Hutchins et al., 2021). Therefore, the primary aim of this research was to observe changes in hepcidin and IL‐6 concentration after exercise in the heat in female athletes.

A secondary aim of the project was to explore gut permeability as a potential mediator of this response. No studies to date have examined the relationship between exercise‐induced increases in gut permeability and iron regulation in athletes. A majority of the cytokines post exercise are muscle‐derived; however, other sources of inflammation may exacerbate or prolong elevated inflammation post exercise. Hepcidin release in response to inflammation is a part of the innate immunity to pathogens (Ganz & Nemeth, 2012). Bacteria require iron to replicate; thus, hepcidin functions to sequester iron from circulation to mitigate bacterial replication (Ganz, 2019; Vargas & Marino, 2016). Exercise can cause small gram‐negative bacteria (endotoxins) to translocate from the lumen of the intestine into systemic circulation, often referred to as “leaky gut” (Chantler et al., 2021), which may enhance the release of inflammatory cytokines.

Previous studies have shown that gut permeability is increased after long exercise bouts, exercise evoking high body core temperatures, or activities involving repeated ground impact exercise (such as running) (Brock‐Utne et al., 1988; Gill et al., 2015; Lambert, 2004). Changes in gut permeability have been attributed to the redistribution of blood flow away from the splanchnic region during exercise and thermoregulatory challenge (Takala, 1996), which can lead to cell damage and increased gut permeability (Van Wijck et al., 2012). Exercise or environmental elevations in body core temperature can compromise the integrity of the protective lining in the intestine (Dokladny et al., 2016). Previous studies have indicated that body core temperatures exceeding 39°C will elicit augmented permeability in exercising individuals (Pires et al., 2017). If an exercising individual attains high body core temperatures, they may be susceptible to larger increases in inflammatory cytokines due to the compounding stimuli of exercise and endotoxins, which may exacerbate hepcidin production, but this is currently unknown.

Only one previous study has explored the connection between endotoxin injection and hepcidin response in humans. Kemna and colleagues demonstrated that injection of lipopolysaccharide (a surface marker on bacteria, or endotoxin; LPS) resulted in a greater inflammatory response and ultimately an increased release of hepcidin with a dramatic 57% decline in circulating iron 22 h after injection (Kemna et al., 2005). While these data confirm the relationship between endotoxin in circulation and hepcidin release, these results need to be contextualized to exercising athletes. It is unknown if athletes experience high enough concentrations of circulating endotoxin to evoke a similar hepcidin response.

The purpose of this study was to examine the time course and magnitude of gut permeability, inflammatory biomarkers, and hepcidin responses evoked by exercise under cool and hot environmental conditions in female athletes. We hypothesized that exercise in a hot environment would increase IL‐6 and hepcidin concentrations post‐exercise compared to a cool exercise condition. We further hypothesized that these elevations are due to the presence of gut endotoxins in circulation.

## 2 MATERIALS AND METHODS

### 2.1 Subjects

Before participating in the study, volunteers provided written informed consent. The study conformed to the principles of the Declaration of Helsinki, except for registration in a database. The research protocol was approved by the Institutional Review Board at the University of Oregon. Fourteen endurance trained female runners (Table 1) completed three exercise visits. All data collection was completed during the winter and spring months in Eugene, Oregon and therefore likely are not influenced by seasonal heat acclimatization. Participants were limited to Tier 2 or Tier 3 athletes as defined by McKay and colleagues (McKay et al., 2022). Seven participants were using contraceptives consisting of five intrauterine devices (one copper, four hormonal) and two oral contraceptive users. Additionally, 10 participants self‐reported regular menstrual cycles. Upon completion of the informed consent, a blood sample was collected to assess iron status (serum iron, transferrin, and ferritin) during a screening visit. Participants were screened and excluded from the study if they exhibited iron status values outside of the normative ranges (transferrin: 188–341 mg/dL; iron, total: 40–190 mcg/dL; ferritin: 16–154 ng/mL).

**TABLE 1:** Participant anthropometrics, exercise speed, and iron status.

Variable	
Age (y)	26.1 ± 5.9
Height (m)	1.65 ± 0.07
Body mass (kg)	62.0 ± 6.7
Body mass index (kg m^−2^)	23.0 ± 2.3
VO_2max_ (mL kg^−1^ min^−1^)	57.7 ± 7.3
Exercise speed (km⋅h^−1^)	10.5 ± 1.3
Exercise grade (%)	1.0 ± 0
Serum iron (mcg/dL)	106.2 ± 53.5
Serum transferrin (μg/dL)	267.4 ± 28.6
Serum ferritin (ng/mL)	32.2 ± 16.5
*n*	13

*Note*: Values are means ± SD.

### 2.2 Study design

#### 2.2.1 Visit 2

Participants completed a graded exercise test to assess VO_2_max on a treadmill at room temperature (21°C–22°C) with a fan on for convective cooling. Participants were asked to abstain from food (3 h), vigorous exercise (24 h), caffeine (6 h), over the counter medications or supplements (12 h), and alcohol (12 h) prior to their graded exercise test. Upon arrival to the lab, height and body mass were recorded, and participants were instrumented with a heart rate monitor (Polar H10, Polar Electro). Participants provided a urine sample to test for hydration status (Atago; Master‐URC/NM, Tokyo, Japan) and pregnancy status (hCG‐Urine Test, Sekisui Diagnostics, San Diego, CA). If urine specific gravity >1.020, participants were asked to drink 5 mL of water per kilogram of body weight. For the graded exercise test, participants were given a standard 3‐min warm‐up (9.66 km/h and 1% grade). Treadmill speed for the test was self‐selected by participants to reflect a 10‐kilometer race pace. After warm‐up, treadmill speed was increased to the testing pace. Treadmill incline was increased 1% per minute until volitional exhaustion. During the test, rating of perceived exertion was collected every minute (Borg Scale). Upon completion of the test, participants were given a 10‐min break before a supra‐maximal VO_2_max verification. Supra‐max validation consisted of a maximal running test set to 110% of the final workload achieved in the graded exercise test. Participants were instructed to run as long as possible, and the supra‐max verification test ended at volitional exhaustion. During exercise, participants wore a mouthpiece, and expired gases were collected and analyzed (Parvo, Parvometrics, Sunvalley, UT). Criteria for a valid VO_2_max test were: a respiratory exchange ratio of ≥1.1, rating of perceived exertion ≥17, a plateau in heart rate/VO_2_ observed despite further increases in workload, and VO_2_ value within 5% of supra‐max validation.

#### 2.2.2 Visits 3 and 4

Prior to arriving at the lab on experimental study days, participants consumed a body core temperature pill at least 4 h prior to exercise (e‐Celsius performance pill, BodyCap, Caen, France or HQ Inc., Palmetto, FL) and maintained a 24‐h food log that was repeated for the subsequent experimental session. Additionally, participants were asked to follow the same abstentions as described for Visit #2. Exercise sessions were conducted at least a week apart and occurred in the morning and in a fasted state to mitigate any potential circadian influence. Menstrual cycle was not controlled for during the experimental sessions. Previous research has examined similar hepcidin responses to exercise across the phases of the menstrual cycle and during oral contraceptive use (Alfaro‐Magallanes et al., 2021). Upon arriving to the lab for the experimental visit days, participants provided a urine sample to test for hydration status (Atago; Master‐URC/NM, Tokyo, Japan) and pregnancy status (hCG‐Urine Test, Sekisui Diagnostics, San Diego, CA). If urine specific gravity <1.020, participants were asked to drink 5 mL of water per kilogram of body mass. Subjects provided a nude body weight before and after exercise to calculate water loss estimates. Visits 3 and 4 were experimental exercise sessions that took place in an environmental chamber. These visits were identical, except for the ambient temperature in the room. One visit was in hot conditions (35°C and 40% relative humidity) while the other was in cool conditions (11°C and 40% relative humidity). The order of the visit conditions was randomized. The exercise duration was 45 min and participants ran at 70% of their VO_2_max. Body core temperature and heart rate were monitored continuously during exercise (Table 2). If a body core temperature of 39.5°C was reached during exercise, the exercise bout was ended.

**TABLE 2:** Measures associated with the exercise bouts.

Variable and timepoint	Cool condition	Hot condition
Heart rate (BPM)		
Start exercise	143 (132, 153)	146 (137, 156)
End exercise	154 (142, 165)^b^	167 (148, 187)^a^, ^b^
Sweat loss (L)		
Post exercise	0.50 (0.41, 0.59)	0.85 (0.73, 0.97)^a^
Body core temperature (°C)		
Start exercise	37.3 (37.1, 37.5)	37.2 (37.0, 37.4)
End exercise	38.2 (38.0, 38.4)^b^	39.2 (38.9, 39.5)^a^, ^b^

*Note*: Values are means (95% confidence intervals).

^a^ Denotes a difference compared to the cool condition at same timepoint.

^b^ Denotes a difference from the pre‐exercise time point within condition.

### 2.3 Blood sampling

Immediately prior to exercise, subjects provided a blood sample via straight needle draw. Immediately post‐exercise, subjects were removed from the environmental chamber and an intravenous catheter was placed for a post‐exercise blood sample and further sampling. Hourly, blood samples were collected for 3 h post‐exercise. One 8 mL serum separator tube was collected at each time point. The blood samples were allowed to clot at room temperature for 30 min before being centrifuged, aliquoted into 1 mL cryovials, and frozen at −80°C for biomarker analysis.

### 2.4 Blood analyses

Iron status (serum iron, transferrin, and ferritin) blood analyses were conducted by an external clinical laboratory (Quest Diagnostics). Lipopolysaccharide was used as the biomarker for leaky gut as this surface protein is expressed on the outside of gram‐negative bacteria. The presence and change in LPS in serum samples was used to indicate changes in gut permeability. Lipopolysaccharide concentration was determined via LAL Chromogenic Endpoint Assay (Hycult Biotech (HIT302), East Hartford, CT). IL‐6, TNFα, and Hepcidin were analyzed using enzyme linked immunosorbent assays (Quantikine HS ELISA Human TNFα (HSTA00E), Quantikine HS ELISA Human IL‐6 (SS600C), Quantikine HS ELISA Human Hepcidin (DHP250); respectively; R&D Systems, Minneapolis, MN). Blood analyses were conducted in duplicate for all subjects at all time points, except one subject missing immediately post exercise and 3 h post exercise timepoints in the cool condition. Out of the 130 sample timepoints for each biomarker that were measured, two are missing due to not getting a blood sample. For specific markers, some samples were excluded as follows: IL‐6; 4 samples over the limit of detection. Hepcidin; 4 samples removed due to high coefficients of variation between tested duplicates. LPS; 10 samples removed due to high coefficients of variation between tested duplicates (7 samples) or samples under the limit of detection (3 samples).

### 2.5 Statistical analyses

Values are expressed as means (95% Confidence Intervals) unless stated otherwise. Alpha was set to 0.05 for all statistical inferences including familywise error rates. Shapiro–Wilk tests were used to assess normality. Fixed effects were condition (hot or cool condition) and timepoint (baseline, 0 h, 1 h, 2 h, and 3 h post exercise). Inferences were drawn from two‐way mixed‐effects models for measurements across time and between conditions (Prism 10, GraphPad, Boston, MA, USA). Inferences regarding changes over time within each condition were drawn from Dunnett's multiple comparison test versus baseline, with reported *p* values and confidence intervals adjusted for multiplicity. Inferences regarding the difference between conditions were drawn from Šidák's multiple comparisons test, restricted to comparisons at the same timepoint between conditions.

### 2.6 Limitations

The primary limitation of this study is that we did not track and match menstrual cycles for the exercise sessions. Since the primary outcome of these research efforts was to observe inflammatory and iron regulatory responses in the heat and was not menstrual cycle specific, we did not match cycle phase. Controlling for menstrual phase would limit the findings to one specific temporal occurrence (i.e., only applicable in the follicular phase) and would limit the ecological validity of the results. We recorded contraceptive type and use across participants as well as self‐reported menstrual cycle status and we did not find any systematic impacts on our data or findings. It is important to note that the menstrual cycle influences resting body core temperature (Baker et al., 2020), however, the starting body core temperatures between the hot and cool exercise bouts were similar (Table 2). Menstrual cycle may also influence gut permeability, however, there is currently a lack of evidence to support this in female athletes (Pugh et al., 2022). Additionally, the use of LPS as a biomarker of gut permeability may present challenges. We followed the manufacturers specifications for performing the assay including using endotoxin free water. However, the appearance of LPS in circulation may be difficult to capture due to the liver and antibodies that neutralized LPS in vivo. Other biomarkers such as intestinal fatty acid binding protein (I‐FABP) or direct markers of gut permeability such as lactulose may be more appropriate for future investigations.

## 3 RESULTS

### 3.1 Exercise

**FIGURE 1:**
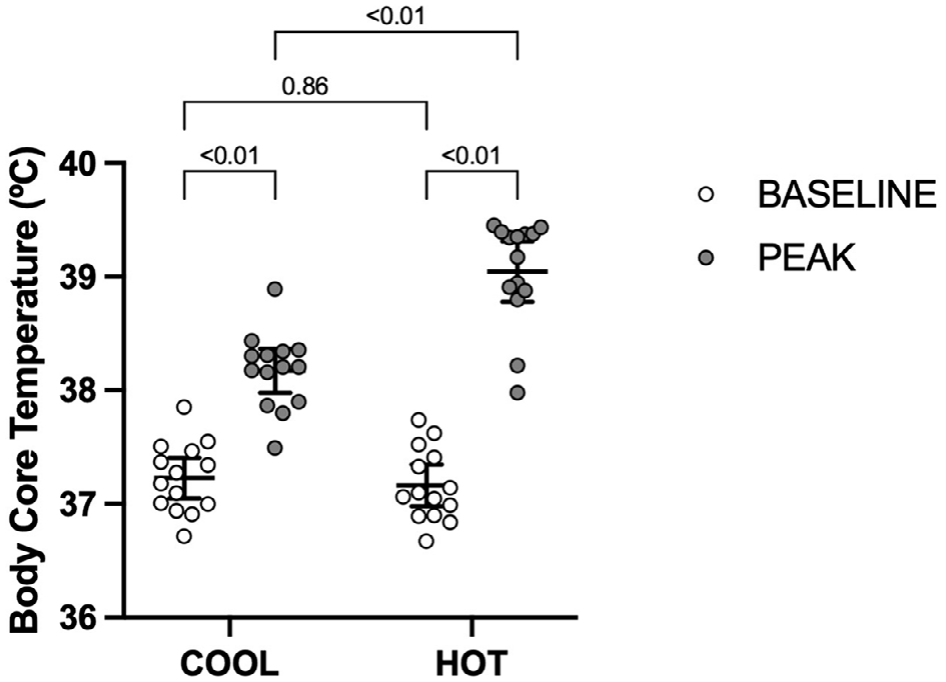
Mean body core temperature (95% CI) during exercise in hot and cool conditions.

Participants completed 45 min of exercise in the cool condition, and average exercising time in the hot condition was ~42 min as five of the exercise sessions were stopped early (four due to body core temperature exceeding 39.5°C and one asked to stop due to lightheadedness). Body core temperature was elevated in both hot and cool conditions after the first 5 min of exercise and this persisted through the end of the exercise session. The rise in body core temperature was greater in the hot condition from minute 25 (*p* = 0.03) until the end of exercise (*p* < 0.01). Peak body core temperature during the hot condition was greater than the cool condition (change +2.1°C (1.8, 2.3), +1.0°C (0.8, 1.3), mean (95% confidence interval), respectively; *p* < 0.01; Figure 1). Peak heart rate and sweat rate were greater in the hot condition compared to the cool condition (*p* < 0.01, *p* < 0.01; Table 2).

### 3.2 Lipopolysaccharide

**FIGURE 2:**
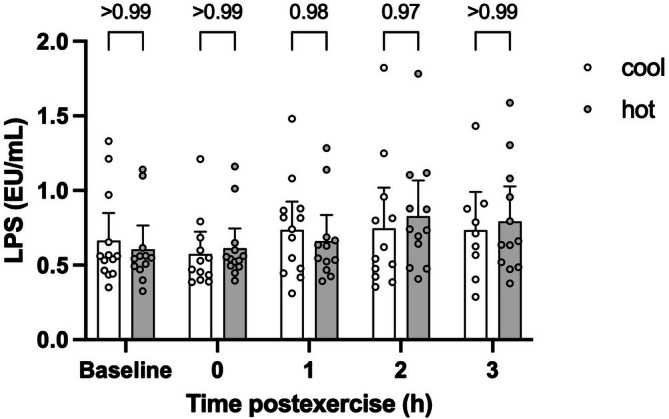
Mean Lipopolysaccharide concentration (95% CI) from pre‐exercise in the hot and cool conditions immediately, 1 h, 2 h, and 3 h post exercise.

Lipopolysaccharide remained at a similar level through the duration of all timepoints and both exercise trials (Figure 2).

### 3.3 Tumor necrosis factor alpha

**FIGURE 3:**
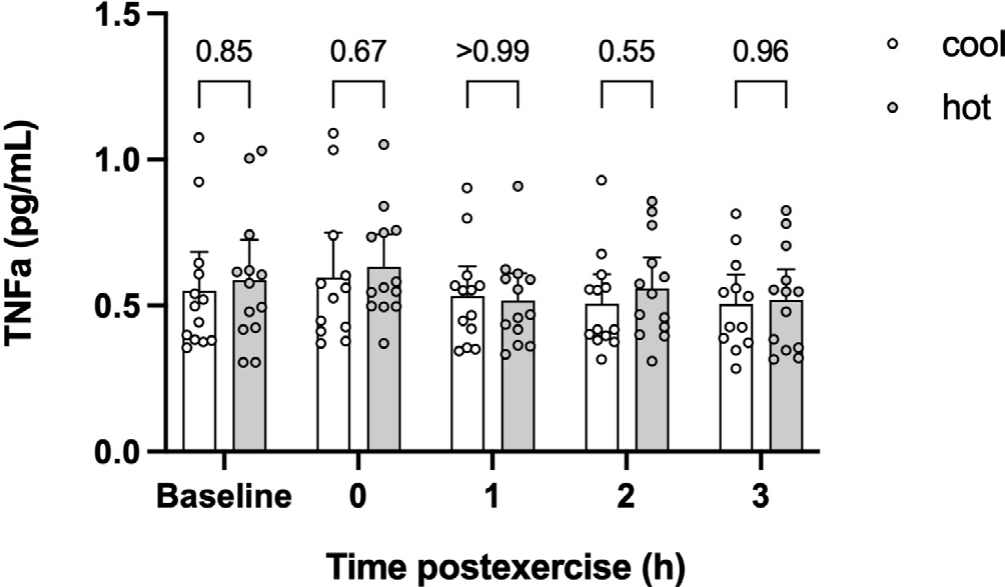
Mean Tumor necrosis factor alpha concentration (95% CI) from pre‐exercise in the hot and cool conditions immediately, 1 h, 2 h, and 3 h post exercise.

TNFα remained at a similar level through the duration of all timepoints and both exercise trials (Figure 3).

### 3.4 Interleukin‐6

**FIGURE 4:**
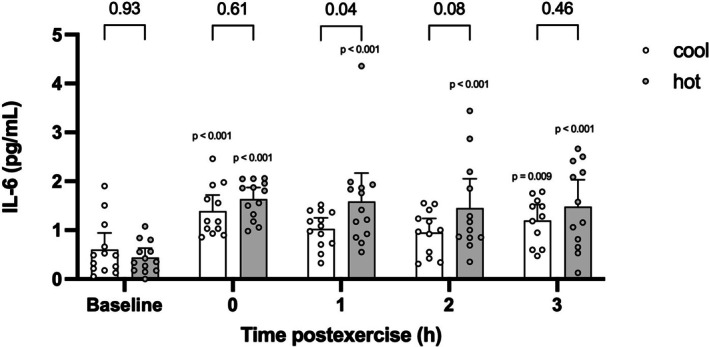
Mean Interleukin‐6 concentration (95% CI) from pre‐exercise in the hot and cool conditions immediately, 1 h, 2 h, and 3 h post exercise. Comparisons were made with a two‐way mixed‐effects model. Values above each bar represent within condition comparisons of each timepoint to baseline. Bracketed values represent between condition comparisons at the same timepoint.

There was a main effect of timepoint (*p* < 0.01) and condition (*p* = 0.05) as well as an interaction between timepoint and condition (*p* = 0.02). IL‐6 increased post exercise in both conditions compared to baseline concentration. IL‐6 was elevated in the cool condition at immediately post (*p* < 0.01) and 3 h (*p* < 0.01) post exercise. IL‐6 was elevated at all timepoints in the hot condition compared to pre exercise (*p* < 0.01; all timepoints). IL‐6 elevations were similar between the two conditions immediately, 2 h, and 3 h post exercise. At 1 h post exercise, IL‐6 concentration was greater in the hot condition (1.6 pg/mL (1.0, 2.2)) versus the cool condition (1.0 pg/mL (0.8, 1.3); *p* = 0.04; Figure 4).

### 3.5 Hepcidin

**FIGURE 5:**
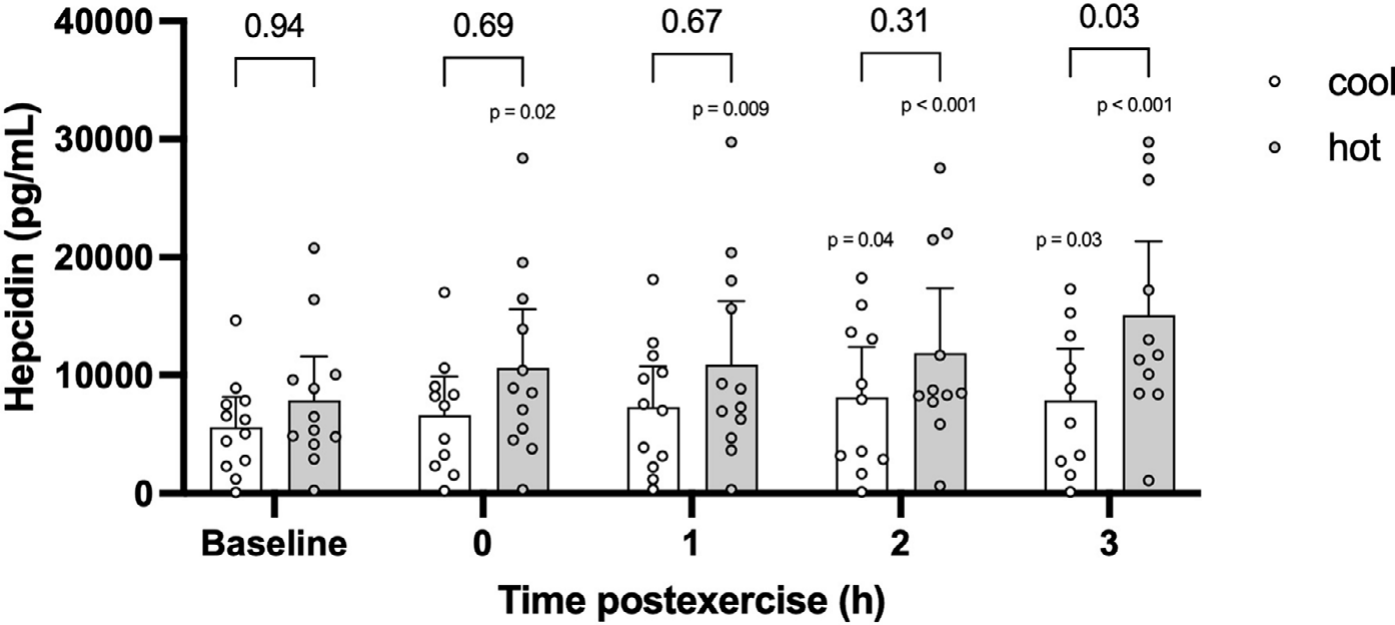
Mean Hepcidin concentration (95% CI) from pre‐exercise in the hot and cool conditions immediately, 1 h, 2 h, and 3 h post exercise. Comparisons were made with a two‐way mixed‐effects model. Values above each bar represent within condition comparisons of each timepoint to baseline. Bracketed values represent between condition comparisons at the same timepoint.

There was a main effect of timepoint (*p* < 0.01) as well as an interaction between timepoint and condition (*p* < 0.01). Hepcidin increased post exercise in both conditions, but the extent of the peak elevation was higher in the hot condition (Figure 5). Hepcidin was elevated in the cool condition at 2 h (p = 0.04) and 3 h (*p* = 0.03) post exercise. Hepcidin was elevated at all timepoints in the hot condition compared to pre exercise (*p* < 0.02; all timepoints). Hepcidin elevations were similar between the two conditions immediately, 1 h, and 2 h post exercise. At the 3 h post exercise timepoint, hepcidin was higher in the hot condition (15.1 ng/mL (8.8, 21.3)) versus the cool condition (7.9 ng/mL (3.5, 12.2), *p* = 0.03; Figure 5).

## 4 DISCUSSION

The purpose of this study was to examine the acute hepcidin, inflammatory and gut permeability biomarker responses elicited by exercise in hot environmental conditions in trained female athletes. We hypothesized that there would be increased gut permeability after exercise in the hot condition that would evoke a greater inflammatory response leading to increased serum hepcidin concentration. The production of inflammatory cytokines such as IL‐6 is integral to the signaling and release of hepcidin from the liver. Due to the known relationship between inflammation and hepcidin, we were curious if other sources of inflammation may augment the IL‐6 signaling and hepcidin release. Our findings demonstrated that hot environmental conditions resulted in changes to IL‐6 and hepcidin concentrations post exercise in the absence of endotoxin in the circulation. The measured endotoxin (LPS) concentration was not elevated in either condition at any time point. IL‐6 was elevated from baseline and sustained in both conditions, but to a greater extent in the hot condition in these trained athletes. Additionally, hepcidin was elevated from pre‐exercise similarly in both conditions; however, the elevation at 3 h in the hot condition was greater than the cool condition. These results indicate that exercise in the heat results in a greater hepcidin response post exercise after an acute heat stimulus in female runners. These findings extend current understanding by demonstrating heat stress as an independent modifier of iron regulation and highlight that seasonality (i.e., hot summer training) may play a more prominent role in regulating hepcidin than previously appreciated.

### 4.1 Gut permeability during exercise

Lipopolysaccharide is a glycoprotein on the surface of gram‐negative bacteria used in both the clinical setting and research investigations as a blood biomarker that indicates an increase in gut permeability (Lambert, 2009; Lim et al., 2009). While basal levels of LPS are present, elevations in LPS would indicate increased bacterial translocation from the lumen of the intestines into circulation. We hypothesized that LPS would be more elevated in athletes after exercising in the heat compared to exercise under cool conditions. However, our results indicate no change in LPS concentration after exercise in either condition. This was not an expected finding, as a previous meta‐analysis indicated gut permeability (via measures of LPS, dual sugar absorbance testing, and intestinal fatty acid binding protein) was augmented in all individuals once a body core temperature of 39°C was reached during exercise (Pires et al., 2017). In our study, all but one participant achieved a peak body core temperature >39°C during the hot condition and all participants stayed below 39°C in the cool visit. We hypothesized that the separation in body core temperature between these two conditions would illicit greater gut permeability in the heat compared to the cool condition. Pires and colleagues' meta‐analysis was completed on a wide range of fitness levels and predominately males, and therefore may not represent the highly endurance trained female athletes included in this study (Pires et al., 2017). Training can evoke physiological adaptations that are protective against gut permeability issues (Lim et al., 2009) and may have led to adaptations in the female athletes to be more resilient to the hot condition exercise bout. This study is one of few assessing endurance‐trained female athletes at a moderate intensity in the heat. These athletes may have adapted to a heating stimulus through their regular training bouts.

Alternatively, an expansion in blood volume due to training can maintain blood perfusion to the splanchnic region for a longer period, thus delaying the hypoxic tissue effects (Van Wijck et al., 2012). Additionally, exercise can make the gut barrier more robust as repeated exercise bouts resulting in elevated body core temperature can change the composition of the enterocytes of the gastrointestinal tract, resulting in a less permeable membrane (Kuennen et al., 2011). Further, immune changes such as increased production of Immunoglobin G (IgG) may function to keep circulating endotoxins at a minimum. Immunoglobin G functions to bind to foreign pathogen associated molecular patterns, such as LPS, mitigating the subsequent inflammatory response. There is research to suggest that trained athletes have higher concentrations of IgG, potentially to combat chronic endotoxin exposure due to exercise (Barberio et al., 2014).

In our study, the average running speed of the visits was 10.5 km/h (range: 8.4–12.6), representing a workload at 70% VO_2_max which was prescribed for 45 min (average of ~42 min in the hot condition). We speculate that the absolute intensity or duration may have been insufficient to cause changes in gut permeability during exercise to evoke an LPS response. While the absolute running intensity was low, perceived intensity of exercise in the heat was quite high (average rating of perceived exertion was “hard” 16 ± 2 at the end of exercise), and several athletes were removed from the heat early (four due to body core temperature exceeding 39.5°C and one asked to stop due to lightheadedness). That said, previous work has highlighted that the duration of exercise may be more predictive of gut permeability than the ambient temperature (Sheahen et al., 2018). Studies utilizing longer exercise bouts have reported changes in gut permeability and may be more successful in evoking changes in gut permeability than ambient temperature (Brock‐Utne et al., 1988). Our results are similar in magnitude to what has previously been characterized post exercise (Yeh et al., 2013), but there is a chance we were underpowered or had too short of a sampling window.

### 4.2 Immune response to endotoxins

Lipopolysaccharide in the systemic circulation can activate TNFα signaling, resulting in an immune‐mediated inflammatory response. Since LPS concentration did not change after exercise, we would anticipate no changes in the production and release of TNFα. Tumor necrosis factor alpha is a proinflammatory cytokine that can be activated in response to an acute infection or trauma. During short exercise bouts, TNFα is not elevated; however, during long sustained efforts, TNFα can be elevated (Cerqueira et al., 2020; Petersen & Pedersen, 2005). The exercise bout in this study was 45 min; therefore, under normal conditions, TNFα concentration will not rise. Our results further indicate that TNFα did not rise during exercise in either exercise condition. These results suggest that the elevated IL‐6 was not immune‐mediated. That is, our data indicated that the source of the IL‐6 release is not from a TNFα activated mechanism, nor LPS induced inflammatory response. The IL‐6 response post‐exercise is likely mediated via primarily muscle‐derived myokine release in response to exercise.

### 4.3 Sources of inflammation post exercise

IL‐6 is an important cytokine released in response to exercise and has direct effects on the production and release of hepcidin. There are many factors that can contribute to elevated IL‐6 in the heat. Exercise intensity (Febbraio & Pedersen, 2002), energy sensing (Pedersen et al., 2001), muscle damage (Fortes et al., 2013), and muscle heating in mouse models (Obi et al., 2017; Welc et al., 2012) have all been shown to independently raise IL‐6 levels systemically in humans and locally in mouse models. IL‐6 was elevated in both conditions, but to a greater extent in the hot condition. This was the anticipated response, as previous work indicates higher intensity exercise elicits a greater rise in IL‐6 (Ostrowski et al., 2000). The additional stimulus of heat resulted in an increased relative exercise intensity during the hot condition (Table 2), which likely resulted in the increased inflammatory response. Reductions in splanchnic blood flow during exercise are associated with increased relative exercise intensity as well as core temperature and are closely associated with the rise in sympathetic activity. Further, the increase in carbohydrate utilization that accompanies exercise in the heat could promote elevated IL‐6 myokine signaling from the working muscle (Febbraio et al., 1994). Precautions were taken to try to mitigate variability in this response. Participants were asked to maintain a consistent diet throughout their duration of enrollment in the study, and food logs were kept and matched 24 h prior to exercise as an attempt to control carbohydrate intake prior to exercise. Therefore, the increase in IL‐6 release after the hot condition was most likely due to the ambient heat exposure and subsequent increase in relative exercise intensity compared to the cool condition.

### 4.4 Iron regulation and inflammation

The release of IL‐6 during exercise in different ambient conditions may be an important consideration for its effects in iron regulation (Nemeth et al., 2004). Hepcidin is known to block cellular export of iron by blocking and degrading the only known iron exporting channel, ferroportin (Ganz, 2006). This process has important implications for the intake of dietary iron. If hepcidin is present in high concentrations, absorption of dietary iron into circulation is more difficult as ferroportin channels are integral to transporting iron into the circulation (Nemeth & Ganz, 2021). Previous research has shown a marked reduction in iron absorption once hepcidin is released (Barney et al., 2022). Our results demonstrate an increase in hepcidin from baseline in both conditions, but to a greater extent in the hot condition at the 3 h timepoint. This is most likely due to the more greatly sustained elevations in IL‐6 during the hot condition. Previous studies examining iron regulation after exercise in hot ambient temperatures reported similar increases in IL‐6 during exercise in hot conditions (McKay et al., 2021). Despite elevations in IL‐6, McKay and colleagues reported no changes in hepcidin production. This study was conducted on a cohort of male subjects exercising at a workload relative to ~55% VO_2_max in similar ambient conditions. The current study examined an all‐female cohort with a higher relative exercise intensity. These key differences may explain the difference in findings. An increase in exercise intensity regardless of sex could explain an elevated hepcidin response as previous research has shown hepcidin responds in an exercise intensity‐dependent manner (Sim et al., 2013). An established sex difference in iron deficiency prevalence and occurrence in athletes would indicate that there may be differences in iron regulation between males and females (Fallon, 2008). There is limited research examining sex differences in the hepcidin response, particularly in highly trained athletes. Work on both male and female cohorts has indicated that 62% of the variability in hepcidin response is due to baseline serum ferritin and serum iron (Peeling et al., 2017). In the current study, individuals outside of the normative range for ferritin, transferrin, and serum iron were excluded. More research is needed to elucidate the sex differences in iron regulation.

## 5 CONCLUSION

An acute bout of exercise in the heat elicited a greater IL‐6 and hepcidin response compared to that in a cool condition in highly trained female athletes. Peak serum hepcidin (3 h post‐exercise) was higher after exercise in a hot environmental condition compared to a cool exercise condition in highly trained female athletes. The greater levels of hepcidin were likely due to a greater increase in IL‐6 after exercise in the heat compared to the cool condition. Lipopolysaccharide and TNFα were not different across timepoints or conditions and likely did not contribute to the elevation in hepcidin in the heat. Since LPS concentration did not change during either exercise session, it appears that the elevation in IL‐6 and hepcidin was independent of gut permeability changes. These results indicate the importance of considering environmental and seasonal impact on iron regulation for training and competing athletes.

## AUTHOR CONTRIBUTIONS

**Kathryn M. Lucernoni:** Conceptualization; data curation; formal analysis; funding acquisition; investigation; methodology. **Samantha J. Chacon:** Conceptualization; data curation; formal analysis; investigation. **Karen Wiedenfeld Needham:** Conceptualization; data curation; formal analysis; investigation; methodology. **John R. Halliwill:** Conceptualization; data curation; formal analysis; investigation; methodology; supervision. **Christopher T. Minson:** Conceptualization; data curation; formal analysis; funding acquisition; investigation; methodology; project administration; resources; supervision; validation; visualization.

## FUNDING INFORMATION

This research was supported by the Wu Tsai Human Performance Alliance & the Joe and Clara Tsai Foundation.

## CONFLICT OF INTEREST STATEMENT

The results of the study are presented clearly, honestly, and without fabrication, falsification, or inappropriate data manipulation. No conflicts of interest, financial or otherwise, are declared by the authors.

## ETHICS STATEMENT

The study conformed to the principles of the Declaration of Helsinki, except for registration in a database. The research protocol was approved by the Institutional Review Board at the University of Oregon.

## Data Availability

Data will be made available upon reasonable request.
